# Developing and validating the Sexual Health Literacy Scale in an Iranian adult sample

**DOI:** 10.1057/s41599-023-01669-z

**Published:** 2023-04-26

**Authors:** Kazhal Rashidi, Peter Watson, Hojjatollah Farahani, Rasol Roshan Chesli, Fatemeh Akhavan Abiri

**Affiliations:** 1 Department of Psychology, Islamic Azad University of Roodehen, Tehran, Iran; 2 MRC Cognition and Brain Sciences Unit, University of Cambridge, Cambridge, UK; 3 Department of Psychology, Tarbiat Modares University, Tehran, Iran; 4 Department of Psychology, Shahed University, Tehran, Iran

**Keywords:** Humanities, Health humanities

## Abstract

The literature has shown that sexual health literacy has limited applicability in many developing countries. The present study, therefore, aimed to develop and examine the validity and reliability of the Sexual Health Literacy Scale (SHLS) among a sample of 595 Iranian university students. The first analysis yielded themes obtained from a qualitative content analysis of the 118-item SHLS scale. Concepts were extracted using the method of latent content analysis (Bengtsson, NursingPlus Open 2:8–16, 2016). 327 initial codes were extracted and main categories (Elo and Kyngäs, J Adv Nurs. 62 (1): 107–115, 2007) or themes (Graneheim and Lundman, Nurse Education Today 24: 105–112, 2004) obtained consisting of the information source, individual barriers, understanding and application, capacity and motivation, damage, skills, sexual rights, and socio-cultural barriers. In the second analysis, the 595 students were randomly split into two groups. An exploratory factor analysis was conducted on the themes derived and quantified in Phase 1. 6 Factors were obtained and found to be consistent in both groups. Criterion-related validity of sexual health literacy was determined by stepwise multiple regression to predict marital satisfaction. The reliability of SHLS was also investigated. The third analysis examined the fit of the 6 factors obtained from the 595 students in the original sample to a new sample of 221 university students using cross-validation via confirmatory factor analysis. We developed and validated a six-factor structure of the Sexual Health Literacy Scale 106 (SHLS-106): factor 1, Sexual Skills; factor 2, Individual Socio-cultural Barriers; factor 3, Sexual Vulnerability; factor 4, Resources to Access Sexual Information; factor 5, Understanding and Application; factor 6, Capacity and Motivation. SHLS-106 shows good test–retest reliability and criterion, incremental and convergent validities. This is the first study to examine the validity and reliability of the Sexual Health Literacy Scale in an Iranian sample. Considering the acceptable validity and reliability of this instrument, the psychometric properties of SHLS-106 need to be further investigated in diverse, more extended samples to clarify the extent of application of this scale in different settings. SHLS-106 can effectively examine sexual health literacy, a dynamic scale in nature influenced by the individual, healthcare system, contextual, and social factors in different cultures.

## Introduction

Sexual health literacy was first coined by Irvine (2004), who explained sexual health and its impact on sexual behaviors. Health behaviors are defined as any activity undertaken for the purpose of preventing or detecting disease or for improving health and well-being (Conner and Norman, 1996). Many researchers are interested in health behavior to grasp both the determinants of health behaviors and the process of health behavior change. Health Behavior Theory (HBT) has become a key route to understanding health behavior. One of the most crucial purposes of HBT is in developing and appraising the interventions targeting public health in individuals and communities (Noar and Zimmerman, 2005).

Cultural values seem to have a major impact on instilling health behaviors (Steptoe and Wardle, 1992). In fact, cultural, social, and family influences shape attitudes and beliefs and therefore influence health literacy. Mass culture as represented by news publishing, advertising, marketing, and the plethora of health information sources are also integral to the social–cultural landscape of health literacy (Nielsen-Bohlman et al., 2004). In this way, the role of culture seems to be important in defining health literacy. Cultural literacy refers to what everyone within a culture ought to know, typically including knowledge of canonical works of high culture and historical events associated with national identity (Hirsch et al., 1988).

Sexual health encompasses the ability to understand and weigh the risks, responsibilities, outcomes, and impacts of sexual actions and to practice abstinence when appropriate. In other words, it involves freedom from sexual abuse, the integration of sexuality into people’s lives, and the derivation of pleasure from it (Satcher, 2001). The World Health Organisation (2010) provides a comprehensive definition of sexual health, i.e. a state of physical, emotional, mental, and social well-being that facilitates having a safe, intimate, and pleasurable sexual experience. In seeking, understanding, and evaluating online sexual health information, Martin (2017) mentioned that gender, sexual identity, stigma, structural factors, and social support are important factors in understanding sexual health literacy in young people. She looked at young people’s sexual health decision-making within various information and support contexts, including school, peers, and social media, and considered what influences those contexts might have on decision-making.

Health literacy is a determinant of health behavior (Harsch et al., 2021), and implies the achievement of a level of knowledge, personal skill, and confidence to take action to improve personal and community health by changing personal lifestyles and living conditions (Nutbeam, 1998; Kickbusch, 2001). Health literacy prepares the tools to pursue healthy alternatives and negotiates the world of health care given the particular context of the individual’s life (Jones and Norton, 2007). High levels of sexual health literacy increase the skill of a person in analysis, judgment, discourse, decision-making, and changes in sexual behavior and empower him or her to improve and maintain their sexual health (World Health Organisation, 2015).

In spite of increasing research in the fields of sexual health and health literacy, “sexual health literacy” has not developed greatly. Nonetheless, some studies have been conducted to openly investigate or measure its different aspects (Reinisch and Beasley, 1991; Jones and Norton, 2007; McMichael and Gifford, 2009, 2010; Guzzo and Hayford, 2012; Graf and Patrick, 2015).

Sexual health literacy, by extension, refers to the ability to understand sexual health information and to act on the information available (Satcher, 2001; Jones and Norton, 2007). Sexual health literacy is considered a very significant issue for all people in society. Although people’s concerns about sexual practices and relationships are not new, the changes in health information in this field have changed the nature of these concerns (Livingstone and Mason, 2015; Parker, 2014). Sexual health literacy, with its dynamic nature, when used in complex situations, might be influenced by individual, social, and contextual factors and health systems (Martin, 2005). Reviewing these studies show that various definitions and perspectives exist towards the “Sexual Health Literacy” concept. It follows from this that sexual health literacy should encompass the level of sexual health knowledge, the capacity and motivations to use it, and the contextual influences to finding, interpreting, and using this information. Consequently, the foundation of sexual health knowledge should be based on the functional, interactive, and critical skills necessary to understand, evaluate and use it in everyday sexual and social contexts.

Jones and Norton (2007) believe that although various definitions of sexual health literacy emphasize having sexual health knowledge, this knowledge might be used in a wide range of contexts and have varying usefulness in different parts of the world. Sexual health literacy, for example, has limited applicability in many developing countries. There are some challenges pertaining to developing countries that could complicate addressing their sexual health literacy (Pillai and Maleku, 2015; Harsch et al., 2021). These challenges include chronic poverty, gender imbalance, unfavorable health conditions, and poor infrastructure. Policy objectives, therefore, have a limited role in developing sexual health literacy in developing countries. Jones and Norton further suggest that definitions of sexual health literacy may be helpful in wealthy countries but have limited applicability in many developing countries.

Recently, Graf and Patrick (2015) examined sexual health literacy with a focus on the influence of various sources of sexual knowledge on safe sex knowledge and risky sexual behaviors among middle-aged and older adults in the USA using a quantitative survey to indicate the relationship between sources of sexual knowledge and risky sexual behaviors. In this study, however, the content and quality of the experiences related to the sources of sexual information of the participants have not been examined.

Sexual health literacy aims to empower individuals in their perceptions and decision-making, and in exercising their sexual health rights. Sexual health rights must be free from discrimination, violence, and coercion. Reviewing the literature demonstrates that gaining an appropriate level of sexual health literacy enables a person’s analysis, judgment, decision-making, and modification of behavior. Sexual health literacy also promotes the capacity to grasp and assess dangers related to sexual health, postpones the first sexual experience, and decreases partners. It also leads to selecting low-risk partners, having a secure sexual experience, appropriating an adequate opportunity to express gender roles correctly, individual sexual health promotion, and, finally, amelioration of familial and social health (Maasoumi et al., 2019). The desired level of sexual health protects individuals from risky behaviors with conversely low sexual health literacy, known as poverty in sexual knowledge (Graf and Patrick, 2015), accompanying an increased probability of a high-risk sexual experience, high rates of sexually transmitted infections (STIs), and unwanted pregnancies (Maasoumi et al., 2019). Vongxay et al. (2019) alluded to the importance of sexual and reproductive health literacy (SRHL) with regard to adolescent pregnancy and assessed this variable via some existing tools which were designed as a measure of sexual health literacy or sexual and reproductive health literacy.

Various studies have shown that sexual health literacy goes beyond knowledge-based sexual health and have highlighted both the need for skills and a range of contextual and structural barriers to sexual health literacy, including social circumstances, economic status, gender discrimination, cultural norms, community and family pressures and anything which can influence sexual practices (Gilbert et al., 2014; Jones and Norton, 2007; McMichael and Gifford, 2010).

In Iran, sexuality is regulated by cultural restrictions and prohibitions due to the country’s traditional and highly religious culture. As in other traditional and patriarchal societies, gender differences exist in sexual socialization, starting from the moment of birth. Girls and boys have different socialization processes as a consequence of gendered rules of sexual behavior. As children, females are expected to learn and apply sexual norms earlier than males, whose deviations are more tolerated. Expression of sexuality in males is more tolerated than that in females. Premarital relationships are religiously banned and nonmarital cross-gender interactions are culturally unacceptable in Iran (Rahbari, 2016). Such differentiated cultural considerations in the context of sexuality in Iran lead to issues such as shame and stigma becoming major barriers to communication, learning, and finally sexual knowledge and sexual health (McMichael and Gifford, 2009) with the need for specific direction toward sexual knowledge issues, especially sexual health literacy.

In order to gain the benefits of sexual health literacy in the quality of one’s sexual life and overcome the disadvantages of a lack of communication leading to poor sexual knowledge, it is considered necessary for policymakers to notice the complications of the health issues faced by members of developing countries like Iran and develop plans to enhance sexual health literacy in the reproductive generation from early adulthood. According to McMichael and Gifford (2009) non-marital sex and sexual health issues are associated with shame and stigma which act as major barriers to communication, learning, and finally sexual knowledge and sexual health in societies such as Iraq, Afghanistan, Burma, Sudan, Liberia, and Horn of Africa countries. A sociological perspective of the development of sexual practices and norms (see DeLamater, 1987), consistent with a life span approach (Baltes, 1987), personal sexual knowledge and behaviors may be best understood within the cultural and historical contexts in which they emerge (Graf and Patrick, 2015).

The assessment of sexual health literacy is considered the initial step in planning policies regarding sexual health literacy. In spite of some studies focused on the concept of sexual health literacy and the importance of considering it to avoid difficulties stemming from poverty in sexual knowledge (McMichael and Gifford, 2009; Martin, 2017; Vongxay et al., 2019; Maasoumi et al., 2019), there is an absence of an appropriate, culture-based tool to measure sexual health literacy.

Accessibility of valid and reliable instruments comprising items related and appropriate to the cultural context of the application makes assessment possible, applicable, and authentic. Although an Iranian article introduced a measure for the assessment of sexual health literacy (Maasoumi et al., 2019), because the orientation of the mentioned paper was based on a quantitative study, selecting the items via reviewing the accessible studies in databases with the keywords “sexual health”, “health literacy”, and “literacy”, the authors of the current study found it proper to design a mixed study to acquire items extracted from a culture-based context to ensure the appropriation of the measure for the members of Iran’s culture. Due to the limited number of instruments measuring sexual health literacy in the world (Martin, 2005) and considering that the level of knowledge, sources of information, socioeconomic status, and cultural context play an important role in the sexual health concept, this research aimed to develop and examine the validity and reliability of the Sexual Health Literacy Scale (SHLS-118) among a sample of Iranian university students.

## Methods

### Item generation

Two hundred items were generated based on the results of the content analysis of the initial pool of items based upon the themes extracted from the content analysis on the university student sample and the semi-structured interviews with the experts. The themes were confirmed by the literature review. At this stage, the researchers generated an initial set of 200 items. These items were in the form of statements on a five-point Likert scale ranging from (1) Strongly disagree, (2) Disagree, (3) No opinion, (4) Agree, (5) Strongly agree. To determine face validity and content validity, the 200-item scale was presented to a panel of experts consisting of 12 psychiatrists and clinical psychologists in the field of sexual health, and they were asked to determine the necessity, relevance, simplicity, and clarity of each item in a similar form for specifying the content validity index (CVI) and content validity ratio (CVR). Based on the Lawshe method, the items with a CVR of >0.8 (Rutherford-Hemming, 2018) and CVI of >0.66 (Polit and Beck 2007; Gilbert and Prion, 2016) were retained as final items, and 82 items were deleted, leaving a total of 118 items in the final form. To determine possible ambiguities in understanding the items, the form was tested on a sample of 20 students and 8 items with literary and writing errors revised. An informed consent form was obtained from all participants.

### Participants and procedure

The target population of this study consisted of undergraduate university students participating in classes at the Azad University of Tehran medical branch in 2019. The purpose of the research was described to them and they were then asked to complete the scales. In this way, it was straightforward for students who were ‘single’ to participate. More than 90 percent of the student participants from the Azad Medical University, who formed the population in this study, were from Tehran, therefore, about 10 percent were from the other provinces. All students gave written informed consent prior to participation. More than 90 percent of participants agreed to take part in the study and completed the scales. Three samples were used for this study and are as follows:

#### Sample 1

650 students were selected using convenience sampling. The sample size was determined by Bentler’s rule (1990) and is based on Hair et al. (2013). 55 of these students were excluded due to problems filling in their responses to questions including giving more than 1 answer to each item on the sexual health literacy scale, not answering at least 20% of the items on the scale, and not completing the questions used to measure content validity of the SHLS-118, therefore, the final sample size was 595 and these were entered into the analysis. They comprised of 391 women and 204 men, Bachelor of Science students (58.36%), Master of Art students (31.28%) and Ph.D. students (10.36%), and they ranged in age from 18 to 45 (*M* = 21.42, SD = 5.12).

#### Sample 2

421 married university students from the population in sample 1 (316 women, 105 men) were selected using the convenience sampling method for assessing incremental validity.

#### Sample 3

221 university students (137 female, 84 male) from sample 1 were selected using the convenience sampling method for doing the confirmatory factor analysis.

#### Phase 1

This research was implemented in 3 phases. In phase 1, the theoretical foundations were reviewed using a critical literature review. Content analysis of the constituent components of sexual health literacy was conducted by interviewing 14 university students, after obtaining their informed consent, using convenience sampling from sample 1 (age mean = 22.34 and SD = 3.83). The purpose of the interviews was to find the other components which are related to sexual health history literacy especially those related to the context and culture in Iran in addition to those we extracted from the literature.

This qualitative research was carried out through semi-structured interviews with these 14 students by 5 experienced psychologists and psychiatrists with a minimum of 5 years of experience in the field of sexual health. The analysis continued until 3 persons produced similar results in the interview whereupon interviewing stopped. To perform content analysis and extract concepts, content analysis using the method of latent content analysis (Bengtsson, 2016) was used. Based on this, 327 initial codes were extracted and eventually main categories (Elo and Kyngäs, 2007) or themes (Graneheim and Lundman, 2004) were obtained which consisted of the information source, individual barriers, understanding and application, capacity and motivation, damage, skills, sexual rights, and socio-cultural barriers.

The purpose of Phase 1 was to discover and quantify the components and themes of sexual health literacy in Iranian culture.

#### Phase 2

In this phase, the themes obtained from Phase 1 were analyzed. The exploratory factor structure, convergent and divergent validity, incremental validity, internal consistency (Cronbach’s alpha), test–retest reliability (obtained over a 2-week interval on a separate 30-case convenience sample taken from sample 1), composite reliability and average variance extracted (AVE) were determined for each of these scales. In this paper, explanatory factor analysis in phase 2 and the later confirmatory factor analysis in phase 3 are used to examine construct validity to assess the network of interrelationships between and among theoretical and observable elements that support a construct (Cronbach and Meehl, 1955). The 595 students were randomly divided into two groups using SPSS-25 software and the second group was used for cross-validation of the exploratory factor analysis.

Exploratory factor analysis was performed using Varimax rotation and principal axis factoring. Parallel analysis was also used to test the number of extracted factors. The parallel analysis determines the number of factors in a dataset more accurately than the eigenvalues of >1 criterion or examining the scree plot of the eigenvalues for breaks or discontinuities (Brown, 2006; Fabrigar et al., 1999). The reason for performing the parallel analysis is finding whether extracted underlying factors are due to chance or not. 1000 random datasets obtained from a random sampling of the 595 students in sample 1 were considered for performing this analysis. Factor retention criteria in the exploratory factor analysis were as follows: (a) Items with factor loadings >0.30, (b) items with cross-loadings <0.3 on additional factors (if more than one factor is interpreted), and (c) Low inter-item correlations obtained by the anti-image correlation matrix (Brown, 2006; Tabachnick and Fidell, 2013).

Criterion-related validity of SHLS-118 was assessed which indicates the effectiveness of a test in predicting an individual’s behavior in specified situations (Anastasi, 1976). Incremental validity relates to the contribution of a test—that is, by the extent to which the use of a test (or any other instrument) brings about an increase in the efficiency of decisions made in a given situation compared to the validity of other methods (Urbina, 2004). The incremental validity of SHLS-118 was also assessed using stepwise multiple regression.

Finally, for determining the convergent validity of this scale, the composite reliability (CR) and average variance extracted (AVE) were calculated. For the discriminant validity, MSV and ASV were calculated. Convergent validity points to a high correlation between the test and other variables with which it should theoretically correlate (Anastasi, 1976). These statistical analyses used SPSS 25 at *α* = 0.05.

In addition, we assessed test–retest reliability (obtained over a 2-week interval on a separate 30-case convenience sample taken from sample 1) for SHLS-118.

The purpose of Phase 2 was to explore the psychometric properties of the SHLS. In particular, our aim was to obtain the exploratory factors of the Sexual Health Literacy Scale, the criterion concurrent validity, convergent validity, and incremental validity of the SHLS, an assessment of the test–retest reliability of the SHLS and the internal consistency of the SHLS.

#### Phase 3

In this final phase, cross-validation of the 6-factor solution derived in Phase 2 was carried out using confirmatory factor analysis (CFA) of the 6-factor structure of the SHLS on sample 3 was investigated via LISREL 8.8. The measures were counterbalanced to control for the order effect.

The purpose of Phase 3 was to examine the fit, via cross-validation, of the 6 factors obtained in Phase 2 on the sample 3 data.

### Measures

The Sexual Knowledge and Attitude Scale (SKAS), Sexual Health Questionnaire, Health literacy Questionnaire (HLQ), and Couple burnout (Tavasoli and Nava, 2017; Nejatian et al., 2021) were used for assessing the criterion-concurrent validity of the SHLS and the Sexual Knowledge and Attitude Scale (SKAS) and Sexual Health Questionnaire were used as predictors in a stepwise multiple regression for determining its incremental validity. Lastly, the Marital Satisfaction scale was used as a criterion variable to validate sexual health literacy (Tavasoli and Nava, 2017; Nejatian et al., 2021). These measures are described individually below.

Sexual Health Literacy Scale (SHLS-118). The scale provided in this study consisted of 118 items in the form of five-point Likert scales, with primary dimensions of information source (9 items), individual barriers (9 items), perception and application (23 items), capacity and motivation (14 items), damage (11 items), skills (31 items), sexual rights (7 items) and socio-cultural barriers (14 items).

Sexual Knowledge and Attitude Scale (SKAS). This scale, developed by Besharat and Ranjbar (2013), includes 30 items in the form of two subscales, Sexual Knowledge and Sexual Attitude, in a five-point Likert scale ranging from strongly disagree to strongly agree. The minimum and maximum score on each of the subscales is respectively 15 and 75. The scale has a total score calculated by the sum of two subscale scores which is between 30 and 150. The validity and reliability of this tool have been investigated in a sample of Iranian undergraduate students from the Universities of Tehran and Science and Technology. The concurrent validity of SKAS was assessed through the correlation between SKAS scores and the Golombok–Rust Inventory of Marital State Questionnaire (GRIMS), the Romantic Relationship Scale (RRS), and the Mental Health Inventory (MHI-28) which demonstrated acceptable validity. The reliability indices were reported in terms of retest coefficients (0.89 for both subscales and 0.88 for the total score) and Cronbach’s alpha (0.91 and 0.88, respectively, for sexual knowledge and sexual attitude). Both validity and reliability were reported as acceptable in the study (Besharat and Ranjbar, 2013).

Sexual Health Questionnaire (SHQ). This questionnaire consists of 33 items in the form of a three-level Likert scale including false, I don’t know, and true. The validity and reliability of this tool are acceptable (Manavipour et al., 2009). Topics in the SHQ include sexual desire, confidence in one’s sexuality, difficulty in having intercourse, and sexual education.

Health Literacy Questionnaire (HLQ). This questionnaire, developed by Montazeri et al. (2014), consists of 33 items in the form of a five-level Likert scale ranging from strongly disagree to strongly agree. The 5-factor structure of the questionnaire (access, reading, understanding, appraisal, and decision) was reported as valid. Reported Cronbach’s alpha coefficients to range from 0.72 (reading) to 0.89 (decision) (Montazeri et al., 2014).

Couple burnout. This instrument is an adopted self-evaluation established by Pines (Pines and Nunes, 2003). This questionnaire includes 21 items divided into two major portions comprising physical fatigue and mental fatigue. The items each comprise a 7-point Likert scale ranging from 1 to 7 in value. A value of 1 point to a lack of experience about the content of the item and 7 demonstrates high experience (Pines, 1996). Internal consistency between variables within the domain was reported between 0.84 and 0.90 (Pines and Nunes, 2003). Cronbach’s alpha of this questionnaire on a sample of 240 Iranians was reported as 0.86 (Mahmoudi et al., 2015).

Marital satisfaction. The Azarin Nathan H. modified marital satisfaction scale includes 8 questions with each comprising an 11-point Likert scale ranging from strongly dissatisfied (1) to strongly satisfied (11). The total score ranges between 8 and 88. Cronbach’s alpha in Iranian samples is acceptable ranging between 0.71 (Heydari, 1998) and 0.90 (Ajeli Lahiji and Reza Zakeri, 2018).

## Results

### Preliminary analysis

Scores, scales, and subscales were assessed to see if they followed a normal distribution. Skewness values of >3 and kurtosis > 10, which could be problematic in the analysis must be transformed (Kline, 2005). The skewness values and kurtosis of the items of SHLS-118 and the other scales and subscales were lower than these limits. The test-retest correlation for SHLS-118 was 0.67, *p* < 0.001.

### Exploratory factor structure of SHLS-118

Exploratory factor analysis was performed on SHLS-118. The data were randomly divided into two groups using SPSS-25 software and the second group was used for cross-validation. There was no significant difference between the demographic variables with all *p*-values > 0.05. In addition, a case-to-parameter ratio of 5:1 existed in both groups to ensure a confident examination of a model (Bentler, 1990).

### Exploratory factor analysis of Group 1

The 595 students in sample 1 were randomly divided into two groups to assess the consistency of the 6-factor solution obtained using the total sample of 595 students. The sample size in the first group was 298 students, which according to the Kaiser–Meyer–Olkin (KMO) measure of sampling adequacy was sufficient (KMO = 0.87). Bartlett’s test of sphericity was significant (*χ*^2^ (6903) = 80,952.84 *p* < 0.001), indicating that the correlation matrix is factorable. The result of the parallel analysis showed that 6 factors should remain. The remaining items should have a factor loading >0.5 (5 items were deleted). The result of the anti-image matrix showed that 12 items were redundant and had to be deleted. Finally, 106 items were retained yielding the SHLS-106.

### Exploratory factor analysis of Group 2

The sample size in the second group was 297 students, which according to the Kaiser–Meyer–Olkin (KMO) measure of sampling adequacy was sufficient (KMO = 0.85). Bartlett’s test of sphericity was significant (*χ*^2^ (6804) = 79,584.64 *p* < 0.001), indicating that the correlation matrix is factorable. The result of the parallel analysis in the second group confirmed the findings of the first group.

### Combined sample

Based on the consistency of the results in groups 1 and 2 exploratory factor analysis was performed with 106 items. The overall results indicated that 106 items explain about 86 percent of the variance. Initial factors obtained from the first analysis showed that socio-cultural barriers were combined with individual barriers, and the sexual rights factor was combined with understanding and application in the factor analysis of groups 1 and 2. Finally, 6 factors remained which were named based on the content of the items: Factor 1, Understanding and Application (29 items), Factor 2, Sexual Skills (25 items), Factor 3, Individual Socio-Cultural Barriers (22 items), Factor 4, Capacity and Motivation (12 items), Factor 5, Sexual Vulnerability (9 items) and Factor 6, Resources to Access Sexual Information (9 items), (Table 1).

**Table 1:** Sexual health literacy (SHLS-106) standardized item-factor loadings: sample 1 (S1) (exploratory factor analysis) and confirmatory factor analysis fitting sample 1 (S1) model to sample 3 (S3).

Factor 1			Factor 2			Factor 3			Factor 4			Factor 5			Factor 6		
Understanding and application			Sexual skills			Individual socio-cultural Barriers			Capacity and motivation			Sexual vulnerability			Resources to access sexual information		
Item	Item factor loading		Item	Item factor loading		Item	Item factor loading		Item	Item factor loading		Item	Item factor loading		Item	Item factor loading	
	S1	S3		S1	S3		S1	S3		S1	S3		S1	S3		S1	S3
1	0.34	0.33	1	0.39	0.38	1	0.41	0.39	1	0.41	0.39	1	0.42	0.41	1	0.33	0.34
2	0.39	0.37	2	0.41	0.42	2	0.33	0.31	2	0.40	0.41	2	0.38	0.40	2	0.39	0.38
3	0.31	0.31	3	0.33	0.32	3	0.39	0.38	3	0.33	0.35	3	0.36	0.35	3	0.41	0.41
4	0.34	0.35	4	0.34	0.35	4	0.32	0.35	4	0.37	0.35	4	0.41	0.39	4	0.32	0.33
5	0.41	0.43	5	0.35	0.36	5	0.41	0.42	5	0.36	0.34	5	0.39	0.38	5	0.36	0.38
6	0.43	0.41	6	0.41	0.39	6	0.42	0.41	6	0.43	0.44	6	0.32	0.34	6	0.42	0.34
7	0.41	0.42	7	0.32	0.33	7	0.33	0.32	7	0.41	0.39	7	0.35	0.34	7	0.37	0.36
8	0.37	0.35	8	0.42	0.43	8	0.41	0.42	8	0.38	0.40	8	0.43	0.41	8	0.32	0.33
9	0.44	0.41	9	0.33	0.31	9	0.42	0.43	9	0.34	0.35	9	0.33	0.32	9	0.33	0.35
10	0.38	0.36	10	0.43	0.42	10	0.39	0.38	10	0.32	0.33	Eigen	Value:1.28		Eigen	Value:1.07	
11	0.51	0.52	11	0.38	0.40	11	0.41	0.42	11	0.37	0.36	Explained	Variance:14.2		Explained	Variance:11.8	
12	0.39	0.38	12	0.39	0.41	12	0.32	0.33	12	0.41	0.43						
									Eigen	Value:1.72							
									Explained	Variance:14.3							
13	0.42	0.43	13	0.41	0.39	13	0.31	0.33									
14	0.41	0.40	14	0.41	0.42	14	0.42	0.43									
15	0.36	0.35	15	0.32	0.35	15	0.37	0.37									
16	0.41	0.41	16	0.43	0.41	16	0.39	0.38									
17	0.46	0.45	17	0.42	0.40	17	0.38	0.37									
18	0.34	0.33	18	0.34	0.36	18	0.41	0.42									
19	0.31	0.32	19	0.37	0.38	19	0.32	0.33									
20	0.35	0.33	20	0.33	0.32	20	0.35	0.34									
21	0.37	0.36	21	0.39	0.40	21	0.41	0.42									
22	0.42	0.41	22	0.41	0.39	22	0.43	0.41									
						Eigen	Value:3.68										
						Explained	Variance:14.72										
23	0.39	0.36	23	0.42	0.41												
24	0.46	0.44	24	0.43	0.44												
25	0.37	0.39	25	0.37	0.35												
26	0.32	0.33	Eigen	Value:3.2													
27	0.33	0.35	Explained	Variance:14.5													
28	0.38	0.37															
29	0.41	0.43															
Eigen	Value:4.41																
Explained	Variance:15.21																

### Phase 2

#### Criterion-related validity

SHLS-106 was positively and significantly correlated with SKAS (*r* = 0.74 *p* < 0.001), positively and significantly correlated with the sexual health scale (*r* = 0.67 *p* < 0.001), positively and significantly correlated with the health literacy questionnaire (*r* = 0.61 *p* < 0.001), and negatively and significantly correlated with couple burnout (*r* = −0.69 *p* < 0.001).

#### Incremental validity

It was determined whether the SHLS-106 would predict marital satisfaction above and beyond the variance accounted for by related measures (i.e., SKAS & SHQ). Results of a stepwise multiple regression on 421 married students indicated that SKAS was entered at step 1 and SHLS-106 was entered at step 2 in the prediction of marital satisfaction (Table 2).

**Table 2:** SHLS-106 incremental variance in marital satisfaction of 421 married students.

	Total *R*^2^	∆*R*^2^	∆*F*	∆*P*
Criterion: Marital satisfaction				
*Step 1*				
SKAS	0.25	0.25	62.17	0.001
*Step2*				
SKAS				
SHLS-106	0.27	0.02	8.91	0.002

Based on Cohen’s criteria (1992), all the values of *r* in the second step had small to medium effect sizes.

#### Convergent validity

CR suggests good reliability when this index is above 0.7 and if the AVE is less than CR and >0.5 then the convergent validity is considered to be confirmed (Gefen et al. 2000). In addition, discriminant validity was established where maximum shared variance (MSV) and average shared variance (ASV) are both lower than the average variance extracted (AVE) (Hair et al. 2013).

Table 3 shows that CR indices are adequately high indicating good reliability for all six factors. In addition, indices of convergent validity indicated no validity concerns; all six factors had an AVE that was less than CR and above 0.5 suggesting convergent validity. All AVE estimates from Table 3 are greater than the corresponding inter-construct squared correlation estimates (MSV and ASV). Therefore, this comparison indicates there is no problem with discriminant validity for the SHLS-106 CFA model.

**Table 3:** The convergent validity and discriminant validity for the SHLS-106 CFA model.

Factor	CR	AVE	MSV	ASV
1-Sexual skills	0.68	0.51	0.34	0.12
2-Individual socio-cultural barriers	0.61	0.55	0.43	0.24
3-Sexual vulnerability	0.65	0.59	0.21	0.11
4-Resources to access sexual information	0.78	0.63	0.03	0.05
5-Understanding and application	0.71	0.56	0.19	0.04
6-Capacity and motivation	0.86	0.68	0.24	0.01

*CR* composite reliability, *AVE* average variance extracted, *MSV* maximum shared squared variance, *ASV* average shared variance.

### Phase 3

#### Confirmatory factor analysis

To examine the confirmatory factor structure of SHLS-106, this tool was used on a sample of 221 university students (120 women and 101 men). We used complete cases and checked for normality as in Phase 2, and eventually, a sample of 205 people remained. Confirmatory factor analysis (CFA) was performed using LISREL-8.8 software, and standard coefficients and model fit indices were determined for SHLS-106 (Table 1).

Model fit was determined via consensus among three indices recommended by Hu and Bentler (1999): the comparative Fit Index (CFI), the standardized root mean square residual (SRMR), and the root-mean-square error of approximation (RMSEA), specifically, CFI values ≥ 0.95, SRMR values ≤ 0.08, and RMSEA values ≤ 0.06 suggest a good fit of the model to the data. Results in Table 4 suggest an acceptable fit. The factor structure obtained in Phase 2 was confirmed in Phase 3 for the students and demonstrated the face validity of the developed scale. In this way, we were able to make sure the items in our questionnaire were measuring what we intended them to measure.

**Table 4:** Model fit indices for the confirmatory factor analyses (CFAs).

*x*^2^/d*f*	CFI	RMSEA	95%CI	SRMR
2.92	0.99	0.064	0.046–0.081	0.02

## Discussion

The main result of this study indicates that the SHLS-106 scale can be used to measure the Iranian cultural and contextual foundation of university students, a population with specific issues requiring sexual health literacy. We assessed the order and content of the factors of this scale. Findings in all three phases in this article show the new measure, SHLS-106 with a 6-factor structure, is valid and reliable in the studied sample. The 6-factor solution has acceptable measures of fit using cross-validation and has good composite, incremental, discriminant, and convergent validities. The SHLS-106 also correlates well with the SKAS and Marital Satisfaction suggesting this is a scale that captures aspects of sexual health literacy.

The dimensions the authors found include Sexual Skills, Individual Socio-Cultural Barriers, Sexual Vulnerability, Resources to Access Sexual Information, Understanding and Application and Capacity and Motivation. Another sexual health literacy scale found in the literature review, Sexual Health Literacy for Adults (SHELA) (Maasoumi et al., 2019) was a 40-item, 4-factor structure with factors “access, reading and understanding, analysis and evaluation, and application”. Regardless of the different methodological designs in SHELA affecting the item numbers and factor structure, the findings of the current research seem to have similarly high convergent validity to SHELA. The difference between the results of SHLS-106 and Sexual Health Literacy for Adults (SHELA) could be due to the difference in ages between the samples of people on which the scales were analyzed. The sample of this study was younger and had fewer married cases than in the SHELA sample.

The social culture in Iran is very important in sexual health literacy because it is related to access to information and to sexual vulnerability with differences related to gender in the Iranian population.

Due to cultural restrictions and prohibitions about sexuality due to Iran’s traditional and highly religious culture, gender differences exist in sexual socialization (Rahbari, 2016) and it is, therefore, necessary to implement culturally based or culturally adapted instruments to measure any aspect of sexuality. Low sexual health literacy, known as poverty in sexual knowledge (Graf and Patrick, 2015), is accompanied by increased probability of high-risk sexual experience, high rates of sexually transmitted infections (STIs), and unwanted pregnancies (Maasoumi et al., 2019).

This tool fills a gap due to the absence of a valid and reliable measure to assess sexual health literacy in different cultures and seems to be applicable in clinical and therapeutic environments to evaluate the extent of cognitive-behavioral sex therapy. This instrument provides a holistic measurement of physical health literacy which is theoretically consistent with the understanding of Nutbeam’s (2000) three-part model and also with the physical health literacy models of Sorensen and colleagues (2013), Jones and Norton (2007), and McMichael and Gifford (2009).

Moreover, in the form of qualitative research, structural and cultural components have been added. The results of this study are consistent with the Dynamic Sexual Health Literacy Model (Martin, 2017) in which sexual health resources, personal issues, and contextual matters are defined in a dynamically balanced pattern therefore, SHLS-106 can effectively examine sexual health literacy, a dynamic scale in nature influenced by the individual, healthcare system, contextual and social factors.

This study aimed to design and introduce a culture-based instrument, the first scale on sexual health literacy in Iran, to assess sexual health literacy. Undoubtedly the primary studies on the foundation and conception of sexual health literacy mentioned by Jones and Norton (2007) and Graf and Patrick (2015) clarified the probable sources of sexual knowledge and its important applications to improving sexual health literacy and the advantages of noticing this concept to avoid results of sexual knowledge poverty. The emerged scale in this study, SHLS-106, tried to improve the limitations of similar scales such as the sexual knowledge and attitude scale and sexual health scale. This instrument has met the requirements for both married and single groups. Following the definition of sexual health literacy, great attention has been paid to sexual skills, capacity and motivation, application, and individual barriers in this instrument. These factors are novel in this developed tool. This study utilized a sufficient sample size for the use of both EFA and CFA as well as cross-validation and reported reliability and validity indices to reflect the appropriate psychometric properties of the newborn scale via a mixed research method.

This scale is usable for assessing sexual health literacy in the culture and context of Iran. This scale is more comprehensive than the Sexual Health Literacy for Adults (SHELA) because the SHLS-106 consists of 6 factors and is more generalizable than the 40-item SHELA which was developed prior to the Coronavirus pandemic in 2019. The incremental validity for the SHLS-106 is higher than that for the SHELA making it a more comprehensive tool for assessing sexual literacy.

The first apparent limitation of the studies in this paper, used to develop SHLS-106, is the use of cross-sectional data collection in a particular period of time. It is impossible, therefore, to extract causality from the relationships reported in this paper. One of the other limitations of this study is non-random sampling and the selecting of participants from university student populations. In particular, the population in this study consisted of medical students mainly from Tehran which may be regarded as having a more liberal culture than smaller more rural areas. These factors make the generalization of findings to clinical and other non-clinical samples difficult and it is anticipated that the findings may be biased due to such limited sampling. The psychometric properties of SHLS-106 in more extended clinical and other non-clinical samples, therefore, need to be further investigated to clarify the extent of application of the mentioned scale in different clinical and research settings.

## Conclusion

The structure of SHLS-106 has acceptable levels of validity and reliability in Iranian university students for measuring sexual health literacy. In particular, we found that the SHLS-106 has a six-factor structure. These results may differ in other populations.

## Data Availability

The participants of this study did not give written consent for their data to be shared publicly, so due to the sensitive nature of the research, and cultural sensitivities, supporting data is not available.
